# The neurobiology of plant-based therapeutics in women's reproductive health: mechanisms, efficacy, and clinical translation

**DOI:** 10.3389/fnut.2025.1591534

**Published:** 2025-05-20

**Authors:** Xue Liu, Chengli Bin, Zehui Zhou, Tongtong Zeng, Kun Wu, Yiping Luo, Qun Liu, Shaobin Wei

**Affiliations:** ^1^Department of Gynecology, Hospital of Chengdu University of Traditional Chinese Medicine, Chengdu, China; ^2^Chengdu Women's and Children's Central Hospital, School of Medicine, University of Electronic Science and Technology of China, Chengdu, China

**Keywords:** neuroendocrine-reproductive axis, plant-derived compounds, women's health, functional foods, phytoestrogens

## Abstract

This review examines the neurobiological mechanisms by which plant-derived compounds influence women's reproductive health through the neuroendocrine-reproductive axis. Gynecological disorders frequently present with neurological manifestations, including cognitive decline in perimenopause, anxiety and depression in polycystic ovary syndrome (PCOS), and central sensitization in endometriosis. Bioactive compounds from medicinal plants, including polyphenols and phytoestrogens, demonstrate therapeutic potential through their anti-inflammatory, antioxidant, and neuromodulatory properties. These multi-target compounds offer advantages over conventional single-target therapies by simultaneously regulating multiple physiological processes. The review explores applications in specific gynecological conditions and discusses the development of dietary supplements and functional foods incorporating these plant-derived ingredients. The growing market for these products presents opportunities for innovative formulations with enhanced bioavailability and personalized approaches. Future research directions include integrating neuroimaging with herbal research, improving clinical translation, and establishing regulatory frameworks for the global application of these plant-derived interventions to enhance female neuroendocrine-reproductive health.

## 1 Introduction

The neuroendocrine-reproductive axis represents a complex and precisely regulated physiological network connecting the central nervous system, endocrine system, and female reproductive system. Proper functioning of this axis is essential for maintaining female reproductive health, emotional stability, and cognitive function (1, 2). With modern lifestyle changes and growing environmental pressures, an increasing number of women experience health issues related to neuroendocrine-reproductive axis dysfunction, including premenstrual syndrome (PMS), polycystic ovary syndrome (PCOS), endometriosis, and menopausal symptoms (3–6).

Epidemiological studies suggest that these gynecological disorders not only impair reproductive function but are also frequently associated with significant neurological symptoms. Studies report that 44% to 62% of perimenopausal women experience cognitive decline, more than 60% of PCOS patients present with anxiety or depression, and 42% of endometriosis patients suffer from chronic pain and central sensitization (7–9). These findings underscore the intricate interplay between neurological and reproductive dysfunctions, emphasizing the need for holistic interventions targeting the neuroendocrine-reproductive axis.

Traditional dietary practices across various cultures have long recognized the unique health benefits of certain plant-based foods for women. These empirical observations have been substantiated by modern nutritional research, demonstrating that bioactive compounds in plant-based foods can modulate neurological and reproductive functions through multiple mechanisms (10–12). Unlike conventional single-target drug therapies, plant-derived compounds typically exhibit multi-target effects, simultaneously regulating multiple physiological processes, making them particularly suitable for addressing complex disorders associated with the neuroendocrine-reproductive axis (13, 14).

**Figure 1:**
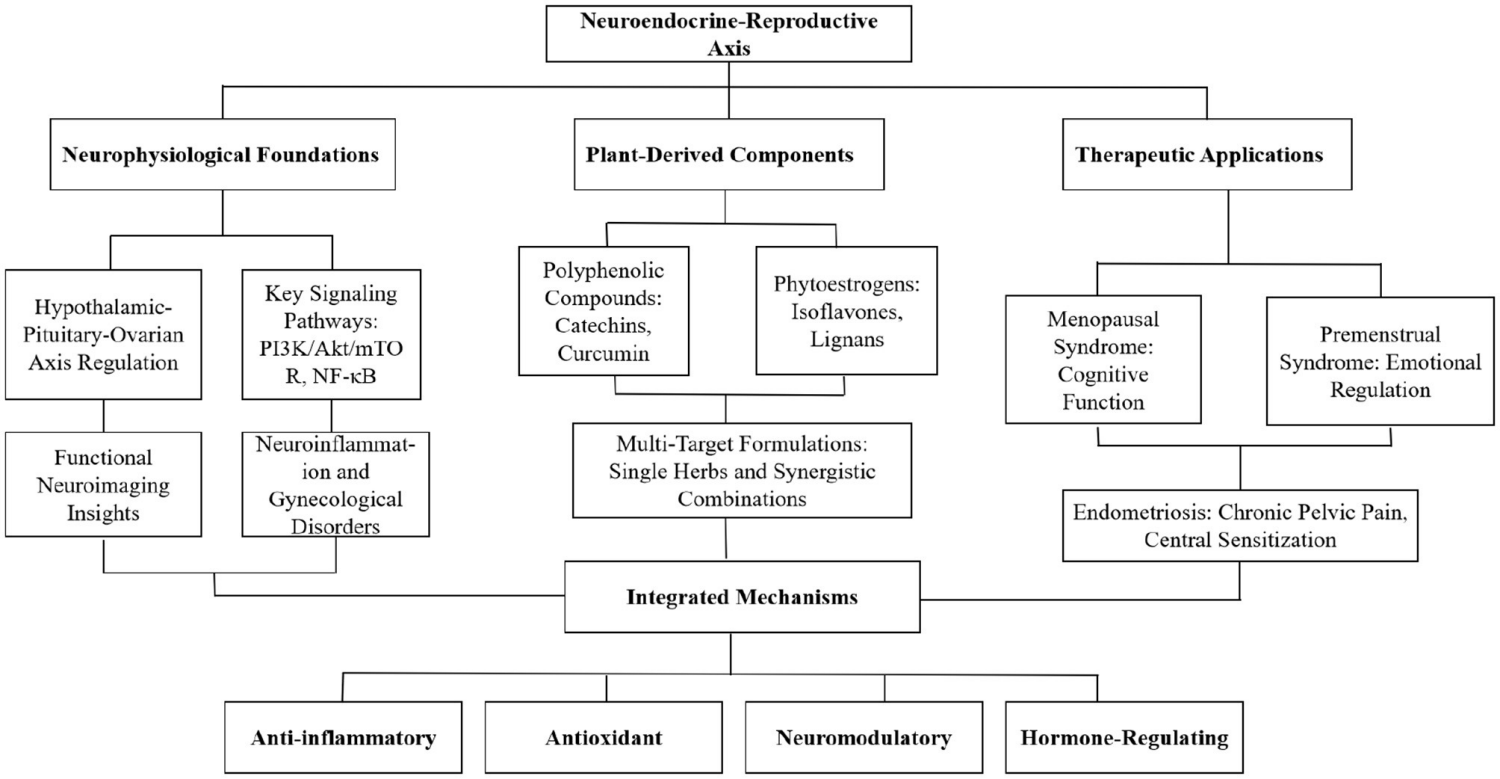
Conceptual framework of the neurobiology of plant-based therapeutics in women's reproductive health.

This review systematically examines the neurobiological mechanisms through which plant-derived compounds influence women's reproductive health via the neuroendocrine-reproductive axis. We analyze the physiological foundations of this axis, explore the mechanisms and biological activities of various plant-derived compounds, evaluate their therapeutic potential in specific gynecological disorders, and propose strategies for their clinical translation. By integrating traditional knowledge with contemporary scientific evidence, we provide comprehensive insights for understanding and utilizing plant-derived therapeutics to enhance female neuroendocrine-reproductive health. The conceptual framework of this review is illustrated in Figure 1, which summarizes the interconnections between plant-derived compounds, their neurobiological mechanisms, and their effects on women's reproductive health.

## 2 Neurophysiological foundations of the neuroendocrine-reproductive axis

### 2.1 Hypothalamic-pituitary-ovarian axis regulation

The hypothalamic-pituitary-ovarian (HPO) axis constitutes the central regulatory network controlling reproductive function throughout a woman's lifespan. This intricate system precisely coordinates the menstrual cycle, follicular development, ovulation, and reproductive aging via tightly regulated hormonal secretion and feedback mechanisms (15). The hypothalamus, serving as the neural command center, secretes gonadotropin-releasing hormone (GnRH) in a pulsatile manner, stimulating the secretion of gonadotropins from the anterior pituitary (16). The subsequently released follicle-stimulating hormone (FSH) and luteinizing hormone (LH) act upon the ovaries to promote follicular growth, ovulation, and the synthesis of estradiol and progesterone (17). Multiple neurotransmitter systems play crucial roles in regulating HPO axis function, particularly the kisspeptin signaling networks in the arcuate nucleus and anteroventral periventricular nucleus (18, 19). These neuronal populations integrate various central and peripheral signals to modulate GnRH pulse release. This neural regulation extends beyond kisspeptin to include dopaminergic, serotonergic, noradrenergic, GABAergic, and glutamatergic inputs, which collectively participate in coordinating reproductive function (20). Evidence from animal and human studies suggests that plant-derived compounds can modulate these neurotransmitter systems, providing a mechanistic basis for their effects on reproductive function (21, 22).

### 2.2 Key signaling pathways in the neuro-endocrine-reproductive axis

**Figure 2:**
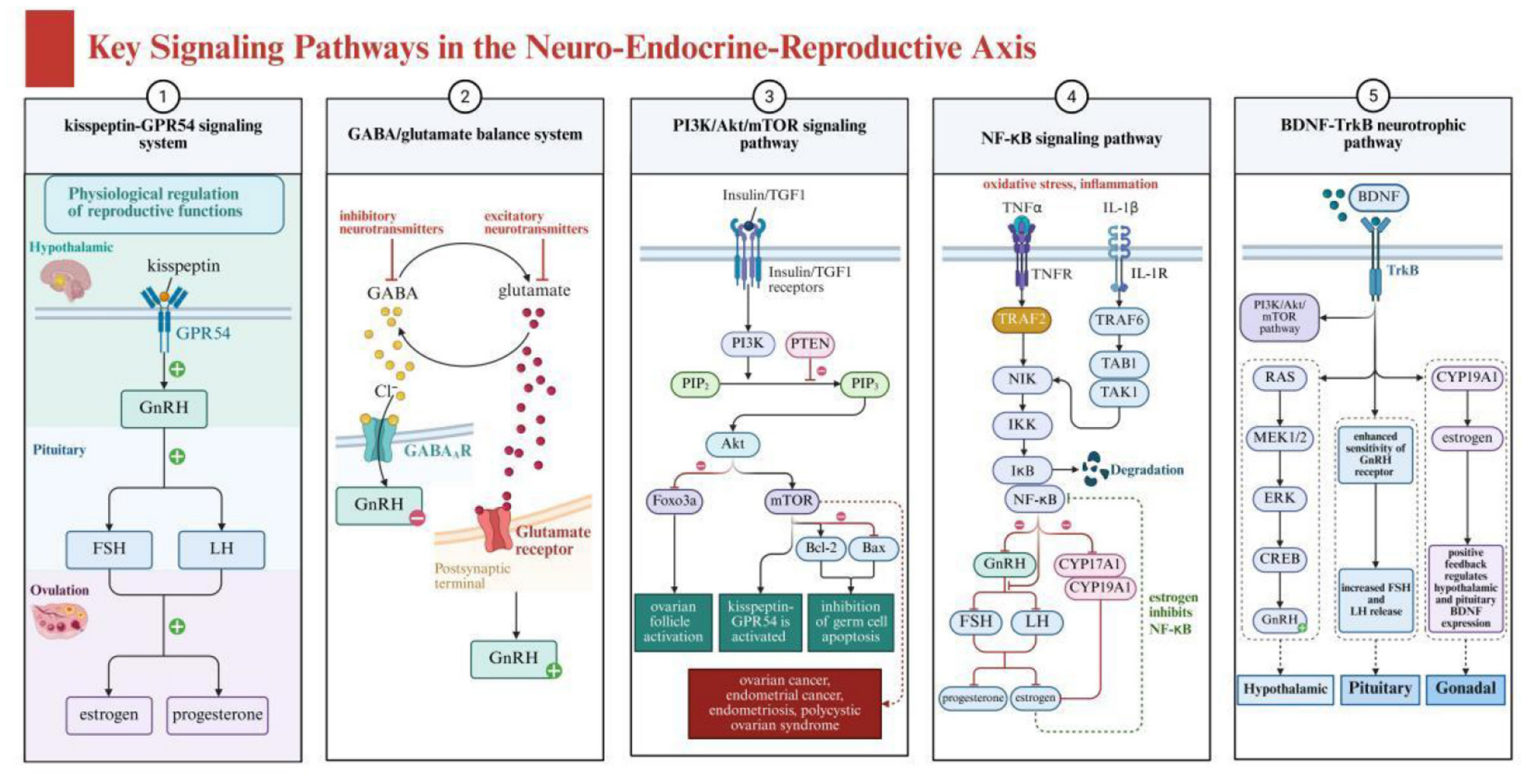
Key signaling pathways in the neuro-endocrine-reproductive axis showing interconnected neural and reproductive regulation systems. Created with BioRender.com.

As shown in Figure 2, the neuroendocrine-reproductive axis involves several key signaling pathways, including the kisspeptin-GPR54 signaling system, GABA/glutamate balance system, PI3K/Akt/mTOR pathway, inflammation-related pathways, and BDNF-TrkB neurotrophic pathway. These pathways collectively form the molecular foundation for the actions of plant-derived compounds. Regulation of the HPO axis involves the precise coordination of multiple signaling pathways, which form the molecular foundation for the actions of plant-derived compounds. The kisspeptin-GPR54 signaling system is particularly critical, functioning as an upstream regulator of the GnRH pulse generator and integrating various endogenous and exogenous signals (23). Kisspeptin neurons activate GnRH neurons through the GPR54 receptor, enhancing their pulsatile release, and research indicates that certain plant flavonoids can influence this process by modulating kisspeptin expression (24, 25).

The GABA/glutamate balance system plays a dual role in HPO axis function regulation. GABA primarily inhibits GnRH neuronal activity through GABA-A receptors, while glutamate promotes activity via N-methyl-D-aspartate (NMDA) and non-NMDA receptors (26). Various active components from traditional Chinese medicine, such as saikosaponins and paeoniflorin, have been found to regulate this balance, indirectly affecting GnRH release patterns (27, 28).

The PI3K/Akt/mTOR signaling pathway plays a crucial role in ovarian function and follicular development, while also being closely related to energy sensing and stress responses in the brain. This pathway is regulated by multiple factors, including insulin, IGF-1, and oxidative stress levels (29). Studies show that various plant polyphenolic compounds such as resveratrol and curcumin can modulate this pathway, simultaneously affecting neuroprotection and ovarian function, achieving multi-level regulation of the neuroendocrine-reproductive axis (30, 31).

These plant compounds, by inhibiting the PI3K/Akt/mTOR pathway, can reduce cell proliferation and promote apoptosis, thus playing a role in cancer treatment (30, 31). Additionally, their antioxidant properties help alleviate oxidative stress, protecting the nervous system and supporting reproductive health. Inflammation-related pathways, particularly the NF-κB signaling pathway, constitute an important bridge connecting the immune system, nervous system, and reproductive system. Chronic inflammation can affect hypothalamic GnRH secretion, pituitary gonadotropin release, and ovarian responsiveness to these hormones through this pathway (32–34). Various anti-inflammatory compounds in traditional Chinese medicine, such as tanshinone and tetramethylpyrazine, can effectively inhibit NF-κB activation, reducing inflammatory interference with the neuroendocrine-reproductive axis (35–37).

The BDNF-TrkB neurotrophic pathway not only plays a role in neuroplasticity and emotional regulation but also participates in local ovarian function regulation (38, 39). Plant active ingredients such as ginkgolide B and ginsenoside Rg1 can enhance BDNF expression and TrkB activation, potentially simultaneously improving neural function and promoting ovarian health, providing a theoretical basis for the application of traditional Chinese medicine components in neuroendocrine-reproductive axis disorders (40, 41).

### 2.3 Functional neuroimaging insights into neuro-reproductive connections

While traditional physiological and molecular studies have established the foundational mechanisms of the neuroendocrine-reproductive axis, modern neuroimaging techniques now provide critical visual evidence of the dynamic interactions between neural and reproductive systems (42, 43). These non-invasive methods are essential for understanding the neurophysiological foundations of the axis, as they reveal real-time functional connections that cannot be observed through conventional research approaches (43, 44).

The development of functional magnetic resonance imaging (fMRI) and related technologies has revolutionized our ability to visualize the neural correlates of reproductive hormone fluctuations (45). These advanced techniques provide unprecedented insights into how the central nervous system and reproductive organs communicate, offering objective visualization of the neuro-reproductive interface that strengthens our understanding of the neurophysiological mechanisms underlying women's reproductive health.

During the menstrual cycle, fMRI studies reveal significant fluctuations in brain activity corresponding to hormonal changes. During the follicular phase, when estrogen levels rise, there is enhanced activation of the prefrontal cortex, improving cognitive control and emotional regulation (46). In contrast, during the luteal phase when progesterone levels peak, there is increased activity in the amygdala and anterior cingulate cortex, areas associated with emotional processing and pain perception (46). These cyclic changes in neural activity patterns demonstrate the profound influence of reproductive hormones on brain function, helping to explain many neuropsychological symptoms experienced by women at specific points in their menstrual cycle.

In women with gynecological conditions, fMRI studies have identified characteristic neural signatures that differ from healthy controls. Women with primary dysmenorrhea show altered resting-state functional connectivity between the anterior cingulate cortex and periaqueductal gray matter, suggesting central sensitization of pain pathways (47). Similarly, endometriosis patients with chronic pelvic pain show increased gray matter volume in pain processing regions and enhanced functional connectivity in pain networks, even during pain-free periods (48). These neuroimaging findings provide objective markers for central nervous system changes in gynecological conditions and suggest potential neural targets for nutritional interventions.

### 2.4 Neuroinflammation and gynecological disorders

Following our understanding of the neuroanatomical pathways and functional connections of the neuroendocrine-reproductive axis, neuroinflammation emerges as another fundamental mechanism that shapes the physiological foundations of this system (32, 49). Inflammatory processes represent not merely secondary consequences but core regulatory elements within the axis, influencing both normal reproductive function and pathological states (50–52). The complex interplay between inflammatory mediators and neuroendocrine signaling constitutes an essential component of the axis's basic neurophysiology, providing critical insights into how the brain-reproductive system communication can be disrupted in various gynecological conditions (51, 52).

Neuroinflammation represents a key pathophysiological mechanism connecting the nervous system and gynecological health. The female reproductive system and central nervous system share common inflammatory signaling pathways, establishing a bidirectional relationship where inflammation in one system can significantly affect the other. Inflammatory factors, including interleukin-1β (IL-1β), interleukin-6 (IL-6), and tumor necrosis factor-α (TNF-α), can cross the blood-brain barrier or activate neural afferent pathways, thereby modulating central nervous system function (53).

In endometriosis, systemic and local inflammation appears to promote central sensitization of pain pathways. Multiple studies have documented elevated levels of inflammatory cytokines in the peritoneal fluid and circulation of women with endometriosis (54, 55). These inflammatory mediators can induce neuroplastic changes in central pain processing networks, evidenced by altered brain activation in pain perception and regulation-related areas shown in fMRI studies. This central sensitization may explain why many women with endometriosis continue to experience pain even after surgical removal of lesions (48).

## 3 Plant-derived nutritional components and their effects on the neuro-endocrine-reproductive axis

**Figure 3:**
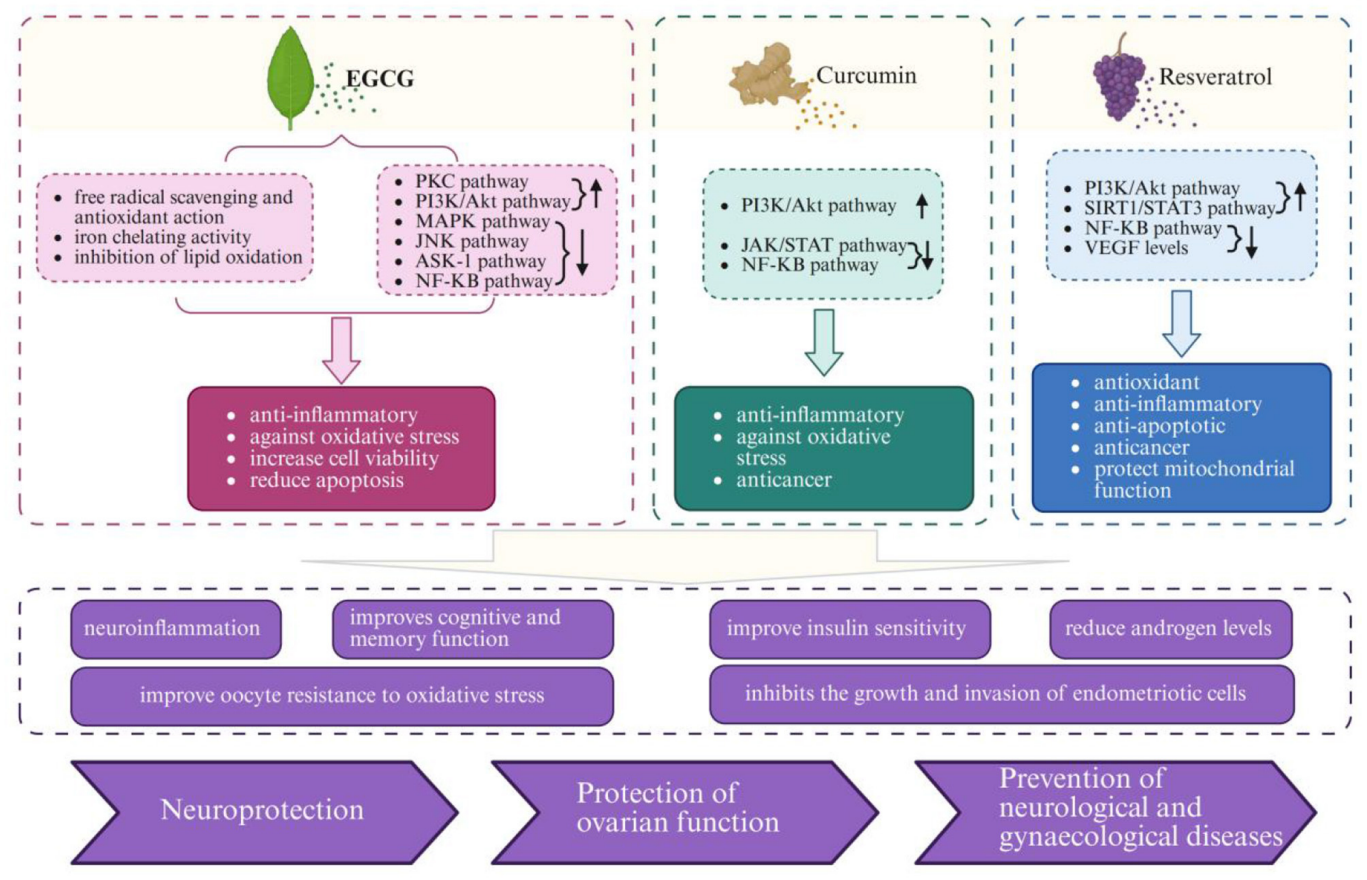
Molecular mechanisms of polyphenolic compounds (EGCG, curcumin, and resveratrol) in neuroendocrine-reproductive health regulation. Created with BioRender.com.

**Figure 4:**
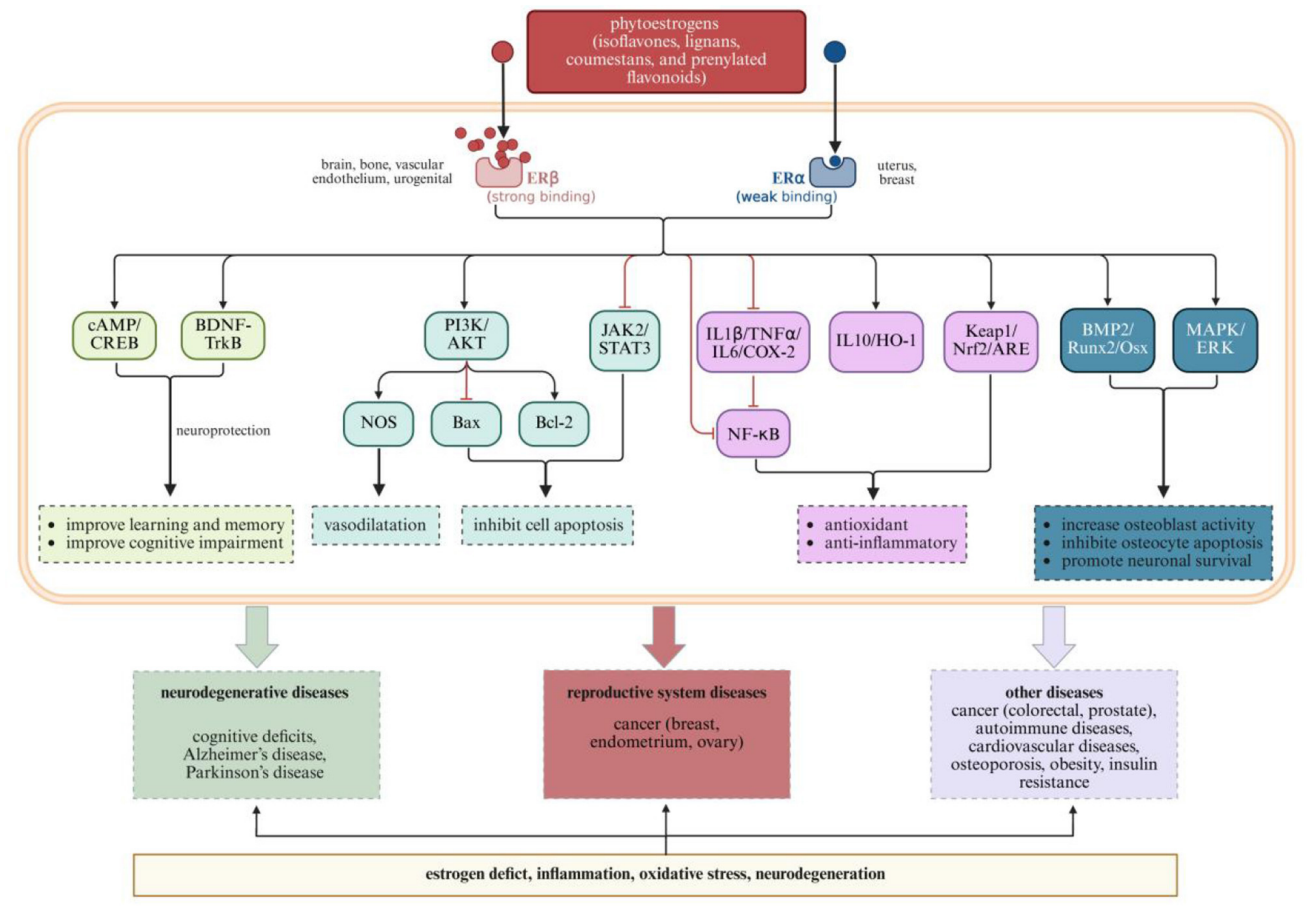
Phytoestrogen action mechanisms and their effects on neurological and reproductive health outcomes. Created with BioRender.com.

As shown in Table 1, Figures 3, 4, different categories of plant-derived compounds, including polyphenols and phytoestrogens, interact with distinct yet overlapping signaling pathways to produce their effects on the neuroendocrine-reproductive axis. Figure 3 illustrates the molecular mechanisms of polyphenolic compounds, while Figure 4 depicts the action mechanisms of phytoestrogens.

**Table 1:** Key plant-derived compounds and their effects on the neuroendocrine-reproductive axis.

**Compound category**	**Representative compounds**	**Main signaling pathways**	**Effects on neural system**	**Effects on reproductive system**	**Clinical applications**
Polyphenols	EGCG (green tea)	PI3K/Akt, NF-κB, PKC, MAPK, JNK, ASK-1	Neuroprotection, Reduces neuronal apoptosis, Anti-inflammatory	Estrogen receptor modulation, Aromatase inhibition, Reduces androgen levels	PCOS, Endometriosis
	Curcumin (turmeric)	NF-κB, PI3K/Akt, JAK/STAT	Anti-inflammatory, Reduces pro-inflammatory cytokines, Protects neural cells from oxidative stress	Inhibits endometriotic cell proliferation, migration and invasion, Downregulates VEGF	Endometriosis, Primary dysmenorrhea
	Resveratrol (grapes)	SIRT1/PGC-1α, PI3K/Akt, NF-κB, VEGF	Suppresses microglial activation, Enhances mitochondrial biogenesis, Reduces pro-inflammatory cytokines	Protects ovarian follicles from oxidative damage, Enhances oocyte quality, Delays ovarian aging through SIRT1 activation	Endometriosis, PCOS, Menopausal mood and cognitive symptoms
Phytoestrogens	Isoflavones (soy)	ERβ-selective binding, BDNF-TrkB, cAMP/CREB, PI3K/AKT	Enhances neuroplasticity, Supports cognitive function, Promotes vasodilation through NOS activation	Selective estrogen receptor modulation, Tissue-specific estrogenic effects	Menopausal cognitive function, Particularly verbal memory and executive function
	Lignans (flaxseed)	Keap1/Nrf2/ARE, IL1β/TNFα/IL6/COX-2, NF-κB	Enhances cellular defense against oxidative stress, Anti-inflammatory, Prevents cognitive deficits	Estrogen receptor binding with different characteristics than isoflavones, Modulates hormone-dependent conditions	Cognitive decline prevention, Hormone-dependent cancers

### 3.1 Polyphenolic compounds

#### 3.1.1 Catechins from green tea

Green tea (Camellia sinensis) contains several catechins, with epigallocatechin gallate (EGCG) being the most abundant and biologically active. EGCG exhibits powerful antioxidant properties through multiple mechanisms: it directly neutralizes free radicals through its hydroxyl groups, demonstrates iron chelating activity, and inhibits lipid oxidation (56–58). These antioxidant actions provide protection against oxidative damage for both neural and reproductive tissues.

EGCG modulates multiple signaling pathways critical to neuroendocrine-reproductive function (59, 60). In particular, EGCG activates the PI3K/Akt pathway, which plays a dual role in neuroprotection and reproductive cell function (60, 61). Within the central nervous system, this pathway activation enhances neuronal survival and reduces apoptosis, contributing to EGCG's cognitive benefits (62, 63). EGCG affects protein kinase C (PKC), MAPK, JNK, and ASK-1 pathways, creating a comprehensive network of neuroprotective mechanisms.

A key mechanism of EGCG's action is its inhibition of the NF-κB pathway, which explains its potent anti-inflammatory effects across both neural and reproductive tissues (64, 65). By suppressing this pathway, EGCG reduces the production of pro-inflammatory cytokines that can disrupt normal hypothalamic-pituitary-ovarian axis function (50, 66). This anti-inflammatory action is particularly relevant for conditions like endometriosis and PCOS, where inflammation contributes significantly to pathophysiology (66, 67).

For gynecological health, EGCG exhibits estrogen-modulating properties through interactions with estrogen receptors and inhibition of aromatase activity (68–71). In PCOS models, these mechanisms help reduce androgen levels, improve insulin sensitivity, and normalize ovarian morphology (66, 72–74). These molecular interactions and their physiological outcomes are illustrated in Figure 3. EGCG's actions culminate in several beneficial outcomes for women's health, including improved insulin sensitivity, reduced androgen levels, and inhibition of endometriotic cell growth and invasion.

#### 3.1.2 Curcumin from turmeric

Curcumin, the principal curcuminoid in turmeric (Curcuma longa), exerts multi-target effects on the neuroendocrine-reproductive axis through several key signaling pathways. Curcumin modulates PI3K/Akt, JAK/STAT, and NF-κB pathways, creating an integrated network of anti-inflammatory and antioxidant actions.

Curcumin's inhibition of the NF-κB pathway is a central mechanism underlying its anti-inflammatory effect (75, 76). By suppressing this pathway, curcumin reduces the production of pro-inflammatory cytokines including TNF-α, IL-1β, and IL-6, which otherwise could disrupt neuroendocrine signaling between the brain and reproductive organs (75, 77). This anti-inflammatory action is particularly important for conditions like endometriosis, where inflammation drives disease progression (77–79).

Curcumin's modulation of the PI3K/Akt pathway contributes to its effects on both neural and reproductive tissues. In neural cells, this modulation enhances survival mechanisms and reduces oxidative stress-induced damage (80, 81). In reproductive tissues like the endometrium, the same pathway modulation helps regulate cell proliferation and angiogenesis, explaining curcumin's beneficial effects in endometriosis (78, 79, 82).

Regarding gynecological health, curcumin demonstrates significant effects on endometriosis through multiple mechanisms illustrated in Figure 3. It inhibits the proliferation, migration, and invasion of endometriotic cells, reduces the expression of inflammatory mediators, and inhibits angiogenesis by downregulating vascular endothelial growth factor (VEGF) (79, 83). These multifaceted actions explain clinical observations where curcumin supplementation significantly reduced the severity of primary dysmenorrhea symptoms compared to placebo (84–86).

#### 3.1.3 Resveratrol from grapes and berries

Resveratrol (3,5,4′-trihydroxy-trans-stilbene) exhibits complex molecular interactions with the neuroendocrine-reproductive axis. It modulates multiple signaling pathways, including PI3K/Akt, SIRT1/STAT3, NF-κB, and VEGF pathways, creating an integrated network of beneficial effects.

A key mechanism of resveratrol's action is its activation of SIRT1, an NAD+-dependent deacetylase that regulates various cellular processes including energy metabolism, stress resistance, and longevity (87–90). SIRT1 activation enhances mitochondrial biogenesis through PGC-1α, improving energy production while reducing oxidative stress (88, 89). This mechanism is particularly important for energy-intensive tissues in the neuroendocrine-reproductive axis, including the hypothalamus and ovaries (90, 91).

Resveratrol's inhibition of the NF-κB pathway explains its anti-inflammatory effects in both neural and reproductive tissues (92, 93). By suppressing microglial activation and reducing pro-inflammatory cytokine production, resveratrol creates a favorable environment for proper neuroendocrine signaling. This anti-inflammatory action may benefit conditions characterized by neuroinflammatory processes, such as the mood and cognitive symptoms associated with PCOS and menopause (94, 95).

For gynecological health, resveratrol's multiple actions converge to protect ovarian function and address conditions like endometriosis. Resveratrol protects ovarian follicles from oxidative damage, enhances oocyte quality, and may delay ovarian aging through SIRT1 activation and inhibition of mTOR signaling (96, 97). In endometriosis models, resveratrol inhibits the proliferation and invasion of endometriotic cells, reduces inflammation, and inhibits angiogenesis, thereby limiting lesion growth and development (98–100).

### 3.2 Phytoestrogens

Phytoestrogens are plant-derived compounds with structural similarities to 17β-estradiol, enabling them to bind to estrogen receptors and produce estrogen-like or estrogen-modulating effects (101). These compounds interact with the neuroendocrine-reproductive axis through multiple signaling pathways, creating tissue-specific effects relevant to women's health.

#### 3.2.1 Isoflavones from soy and red clover

Isoflavones, primarily daidzein, genistein, and glycitein, interact with the neuroendocrine-reproductive axis through their preferential binding to estrogen receptor β (ERβ) over estrogen receptor α (ERα), with binding affinities for ERβ ~17–324 times higher than for ERα (102). This selective receptor binding property explains their tissue-specific effects, as ERβ is predominantly expressed in the brain, bone, and vascular endothelium, while ERα is more abundant in reproductive tissues like the uterus and breast.

This selective estrogen receptor modulator (SERM) activity allows isoflavones to exert beneficial estrogenic effects in certain tissues while minimizing unwanted stimulation in others. In the brain, ERβ activation by isoflavones triggers neuroprotective pathways including BDNF-TrkB and cAMP/CREB signaling, which enhance neuroplasticity and support cognitive function (103, 104). This activation improves learning, memory, and cognitive function—effects particularly relevant for menopausal women experiencing cognitive changes.

Isoflavones also modulate PI3K/AKT signaling, which influences both neuronal survival and reproductive cell function (105–107). In neural tissue, this pathway activation enhances cell survival mechanisms and promotes vasodilation through nitric oxide synthase (NOS) activation (108). These effects may explain the observed benefits of isoflavone supplementation on cognitive function and cerebrovascular health in menopausal women.

#### 3.2.2 Lignans from flaxseed and whole grains

Lignans are diphenolic compounds found in various plant foods, with flaxseed being the richest dietary source (109). Lignans interact with the neuroendocrine-reproductive axis through multiple pathways following their conversion to bioactive enterolignans by intestinal bacteria (101, 110–112). These compounds, like isoflavones, bind to estrogen receptors but with different binding characteristics, producing distinct biological effects.

Lignan metabolites modulate multiple signaling cascades beyond direct estrogen receptor binding. They activate antioxidant pathways including Keap1/Nrf2/ARE signaling, which enhances cellular defense against oxidative stress in both neural and reproductive tissues (113). Additionally, they have an inhibitory effect on inflammatory pathways including IL1β/TNFα/IL6/COX-2 and NF-κB signaling, creating an anti-inflammatory environment conducive to proper neuroendocrine function.

These molecular actions culminate in prevention of various conditions affecting women's health, from cognitive deficits to hormone-dependent cancers. For the nervous system, lignans help prevent cognitive deficits and neurodegenerative conditions through their combined antioxidant and anti-inflammatory actions. For the reproductive system, these same mechanisms help address hormone-dependent conditions including reproductive system cancers. This multi-system protective effect exemplifies the integrative approach of phytoestrogens in supporting the neuroendocrine-reproductive axis.

### 3.3 Formulation approaches and multi-target mechanisms

#### 3.3.1 Single plant extracts with multi-target properties

Many single Chinese medicinal plants exhibit multi-target therapeutic effects, showing significant advantages particularly in neurological health (114). The complex chemical composition and multiple bioactivities of Chinese herbal components enable them to target various disease pathways, producing synergistic effects (115). For example, flavonoid compounds in Scutellaria baicalensis possess significant antioxidant and anti-inflammatory actions, improving cognitive function and slowing neurodegeneration progression by inhibiting neuroinflammation and improving blood-brain barrier permeability (116, 117). Polysaccharides, carotenoids, and flavonoids in Lycium barbarum have antioxidant and anti-aging properties, protecting neurons and improving memory and cognitive function by regulating the immune system and enhancing neurotrophic factors (118). Active components in Salvia miltiorrhiza, such as salvianolic acids, demonstrate anti-inflammatory, antioxidant, and circulation-promoting effects, helping improve cerebral blood flow and oxygen supply, reducing neurodegeneration, and showing extensive application potential in treating Alzheimer's disease (119).

#### 3.3.2 Synergistic effects in complex herbal formulations

Chinese herbal formulations embody the principle of synergy, whereby combinations of herbal components work collectively to enhance therapeutic efficacy, minimize adverse effects, and target multiple pathological pathways simultaneously (115, 120). Traditional Chinese herbal formulation follows the “Jun-Chen-Zuo-Shi” (monarch-minister-assistant-courier) principle, where primary herbs address the main pathology (Jun), supporting herbs enhance the primary action (Chen), adjuvant herbs reduce side effects or target secondary symptoms (Zuo), and courier herbs direct the therapeutic action to specific organs or tissues (Shi). This structured approach ensures systematic synergy throughout the formulation process, maximizing therapeutic outcomes while minimizing adverse effects.

Research demonstrates that herbal ingredients within a formulation can potentiate each other's bioactivity through several synergistic mechanisms, including pharmacokinetic synergy where one herb enhances the absorption or bioavailability of another (121, 122). For instance, piperine from black pepper has been shown to increase the bioavailability of curcumin by up to 2,000%, inhibiting hepatic and intestinal glucuronidation and slowing intestinal transit time. This pharmacokinetic enhancement significantly improves the therapeutic potential of herbal combinations compared to individual components (121, 122).

For example, combining tetramethylpyrazine from Ligusticum chuanxiong with astragaloside IV from Astragalus membranaceus significantly enhances neuroprotective outcomes in spinal cord injury by modulating astrocyte polarization. Tetramethylpyrazine exerts anti-inflammatory and neuroprotective effects via NF-κB inhibition, while astragaloside IV potentiates these effects by activating SIRT1 pathways, together creating a robust neuroinflammatory control mechanism superior to either component alone (123–128).

Similarly, the combination of salvianolic acid IIA (from Salvia miltiorrhiza) and tetramethylpyrazine nanoemulsion exhibits synergistic neuroprotective effects in Alzheimer's disease models. Salvianolic acid IIA independently possesses strong antioxidant and anti-apoptotic effects by targeting MAPK/ERK/CREB pathways, while tetramethylpyrazine enhances blood circulation and reduces neuronal inflammation (129). Their co-delivery in nanoemulsion form significantly enhances bioavailability, improving therapeutic outcomes related to cognitive impairment and neuronal survival beyond what single ingredients achieve independently.

The Yangyin Tongnao Granules (YYTN), a complex formulation including formononetin, calycosin, ligustrazine, puerarin, and ferulic acid, exemplify multi-component synergy. This formulation simultaneously targets inflammatory pathways (reducing TNF-α), oxidative stress (elevating T-SOD levels), and apoptosis (lowering Cyt-C levels) in cerebral ischemia-reperfusion injury models. Individually, each herbal component has specific biological activities, but their coordinated interaction within YYTN achieves enhanced neuroprotective efficacy through combined modulation of inflammatory, oxidative, and apoptotic pathways (130).

In women's reproductive health specifically, the traditional formula Gui Zhi Fu Ling Wan demonstrates remarkable synergistic effects in treating endometriosis and dysmenorrhea (131, 132). This formula combines Cinnamomi Ramulus (Gui Zhi) as the monarch herb providing analgesic effects, Poria (Fu Ling) and Moutan Cortex (Mu Dan Pi) as minister herbs enhancing blood circulation and resolving blood stasis, with Persicae Semen (Tao Ren) and Paeoniae Radix (Chi Shao) as assistant herbs that further promote blood circulation while modulating inflammation (133, 134). The combined action of these herbs creates a comprehensive therapeutic effect that simultaneously addresses pain, inflammation, and tissue remodeling in endometriotic lesions, with clinical studies showing superior efficacy compared to single-herb treatments (132, 133, 135).

Additionally, Kai Xin San (KXS), a traditional herbal formula, demonstrates how herbal synergy operates through a multi-pathway approach in Alzheimer's disease. Its combination of herbs collectively enhances cognitive and memory functions by activating the Wnt/β-catenin signaling pathway, facilitating mitochondrial autophagy, and suppressing neuroinflammation via NLRP3 inflammasome inhibition. These coordinated actions provide comprehensive therapeutic effects against neuropathological processes, illustrating the unique strength of synergistic formulations in complex neuroendocrine-reproductive conditions (136, 137).

Therefore, understanding and leveraging the synergistic interactions between herbal ingredients within traditional formulations can significantly optimize their therapeutic potential, providing enhanced efficacy and broader clinical applications for managing complex women's reproductive health disorders.

## 4 Applications of plant-derived nutritional components in specific gynecological conditions

### 4.1 Menopausal syndrome and cognitive function

The menopausal transition marks a significant phase in a woman's life, characterized by declining ovarian function and hormonal fluctuations, ultimately leading to cessation of menstrual cycles. Beyond reproductive changes, menopause is frequently accompanied by a range of symptoms affecting multiple body systems, including vasomotor symptoms (hot flashes, night sweats), psychological disturbances (mood fluctuations, anxiety, depression), cognitive changes (“brain fog,” memory decline), and urogenital symptoms (vaginal dryness, urinary issues) (138). The neurological dimension of menopausal syndrome is increasingly recognized as a significant contributor to declining quality of life and functional capacity. Menopausal estrogen decline affects various neurotransmitter systems, cerebral blood flow, and neuroplasticity, potentially leading to cognitive changes and psychological symptoms (139). Additionally, sleep disruption due to vasomotor symptoms may exacerbate cognitive difficulties and mood disorders, creating a cycle of symptoms that significantly impacts daily functioning (140).

#### 4.1.1 Research on traditional Chinese herbal applications in menopausal cognitive impairment

Various plant-derived compounds found in traditional Chinese herbs demonstrate potential value in improving cognitive function during menopause, particularly those with phytoestrogenic activity. Literature reviews indicate that multiple Chinese herbal extracts can influence cognitive function through multiple mechanisms, including antioxidant, anti-inflammatory, and neuroprotective effects, which are closely related to cognitive changes resulting from declining estrogen levels during menopause (141).

Studies show that flavonoid compounds from Scutellaria baicalensis provide protection to neural cells by regulating oxidative stress and neuroinflammatory processes, mitigating cognitive decline in menopausal women. These compounds not only reduce free radical production but also enhance neuroplasticity through regulation of specific signaling pathways, thereby supporting cognitive functions such as memory and attention (117). Additionally, since Scutellaria extracts modulate activity in the prefrontal cortex, an area that commonly shows decreased activity in menopausal women, they may have specific beneficial effects on executive function (141–143).

Soy isoflavones, as a major source of phytoestrogens, have been extensively studied for their role in improving menopausal cognitive function. Systematic reviews suggest that isoflavones may provide cognitive protection through selective binding to estrogen receptor β (ERβ), which is highly expressed in cognitive-related brain regions such as the hippocampus and prefrontal cortex (144–146). Research has observed that long-term supplementation with soy isoflavones may positively affect verbal memory and executive function, particularly in late-stage postmenopausal women (144, 147, 148). Some research findings indicate that isoflavone intervention may slow the trend of hippocampal volume atrophy in menopausal women and enhance functional connectivity between the hippocampus and prefrontal cortex, with these structural changes correlating with cognitive improvements (149, 150). However, it is worth noting that heterogeneity exists between different studies, suggesting that individual factors, dosage, and intervention duration may influence intervention effects (144).

Traditional Chinese herbal formulations, such as the Ningshen mixture, have demonstrated synergistic advantages in treating menopausal cognitive function (151). Observational studies suggest that these formulations not only alleviate vasomotor symptoms such as hot flashes but also improve cognitive test performance, particularly in memory and attention domains. Mechanistically, these formulations may function by regulating the expression of neurotrophic factors and reducing neuroinflammation (152–154). Compared to single-component interventions, Chinese herbal formulations can simultaneously act on multiple targets, potentially providing more comprehensive regulation for menopausal syndrome symptoms.

#### 4.1.2 Advantages of multi-target mechanisms of Chinese herbal medicine in menopausal symptom management

Unlike single-target Western medications, active components from traditional Chinese medicine exhibit multi-target regulatory characteristics for menopausal syndrome. For example, tanshinone IIA not only selectively activates ERβ and demonstrates estrogen-like effects but simultaneously inhibits inflammatory factor (IL-1β, TNF-α) release, activates the Nrf2 antioxidant pathway, and enhances BDNF expression (155–157). This multi-target characteristic enables a single Chinese herbal component to simultaneously improve vasomotor symptoms, mood fluctuations, and cognitive decline, providing a comprehensive solution for menopausal women.

Modern neuroimaging research has revealed the regulatory mechanisms of Chinese herbal components on menopausal brain function. A study applying electroencephalography (EEG) and functional near-infrared spectroscopy (fNIRS) demonstrated that honeysuckle extract could restore alpha wave activity in the prefrontal cortex of menopausal women and enhance blood oxygen levels in this region, changes significantly correlated with improved cognitive test scores (158). Another study using resting-state fMRI found that ginsenoside Rg1 could enhance functional connectivity in the default mode network of menopausal women, closely related to emotional regulation and self-referential processing, potentially explaining its antidepressant effect (159–161).

In clinical practice, menopausal symptoms often present with individualized differences, requiring personalized intervention strategies (162). Traditional Chinese medicine emphasizes syndrome differentiation and treatment, selecting appropriate herbal formulations based on women's specific symptom combinations, an approach highly consistent with modern precision medicine concepts. Recent research confirms that personalized Chinese herbal interventions based on traditional Chinese medicine constitution typing are superior to standardized prescriptions in improving menopausal symptoms, especially for complex patients with concurrent cognitive and emotional symptoms (162–165).

### 4.2 Premenstrual syndrome and emotional regulation

Premenstrual syndrome (PMS) encompasses a range of physical, emotional, and behavioral symptoms occurring during the luteal phase of the menstrual cycle and disappearing shortly after menstruation begins (166). A more severe form, premenstrual dysphoric disorder (PMDD), is characterized by significant mood disturbances that substantially impact functioning. The pathophysiology of PMS/PMDD involves complex interactions between ovarian hormones, neurotransmitter systems, and neuroendocrine pathways, providing multiple targets for herbal nutritional interventions.

#### 4.2.1 Chinese herbal regulation of serotonergic pathways to improve PMS emotional symptoms

Serotonergic dysfunction occupies a central position in PMS/PMDD pathology, with affected women showing altered serotonin function and increased sensitivity to normal hormonal fluctuations (167). Various active components from Chinese herbs can influence serotonergic neurotransmission, potentially alleviating mood disorders associated with PMS/PMDD (168).

St. John's wort (Hypericum perforatum) contains multiple bioactive compounds, including hypericin and hyperforin, which inhibit serotonin reuptake, potentially enhancing serotonergic neurotransmission (169, 170). A randomized controlled trial found that St. John's wort extract (900 mg daily) significantly reduced physical and behavioral symptoms of PMS, with improvements particularly notable for mood disturbances (171). These effects were comparable to selective serotonin reuptake inhibitors (SSRIs), the first-line pharmacological treatment for PMS/PMDD (172).

Molecular mechanism studies indicate that Chinese flavonoid compounds such as saikosaponins from Bupleurum and apigenin from celery can regulate serotonergic function through multiple pathways. These compounds inhibit monoamine oxidase (MAO) activity, modulate serotonin receptor sensitivity, and influence neurotransmitter reuptake. For example, in PMS animal models, saikosaponin treatment significantly increased serotonin levels in the hypothalamus and hippocampus while upregulating 5-HT1A receptor expression, closely related to antidepressant and anxiolytic effects (173). Functional magnetic resonance imaging (fMRI) studies provide insights into how Chinese herbal compounds affect emotional regulation neural circuits in women with PMS (174).

#### 4.2.2 Anti-inflammatory Chinese herbal components and PMS symptom reduction

Inflammatory processes appear to be important contributing factors to PMS symptoms, with affected women showing elevated inflammatory markers during symptomatic luteal phases (175). Various plant-derived anti-inflammatory compounds may help alleviate these inflammatory aspects of PMS/PMDD.

Alpha-linolenic acid (ALA) from flaxseed and other plant-derived omega-3 fatty acids demonstrate significant anti-inflammatory properties by reducing pro-inflammatory eicosanoid production and inducing specialized pro-resolving mediators (176, 177). A randomized controlled trial found that omega-3 supplementation significantly reduced physical and psychological symptoms of PMS, with particular improvements in depression, anxiety, and bloating (178). These benefits were associated with reductions in inflammatory markers, suggesting a mechanistic link between anti-inflammatory actions and symptom improvement.

Curcumin from turmeric is an effective anti-inflammatory compound that inhibits NF-κB signaling and reduces pro-inflammatory cytokine production (179). Clinical research shows that curcumin supplementation (100 mg every 12 h from 7 days before menstruation to 3 days after) significantly reduces PMS symptoms, with improvements in both physical and behavioral symptoms. These effects were comparable to non-steroidal anti-inflammatory drugs (NSAIDs), suggesting that curcumin may benefit the inflammatory aspects of PMS (180).

### 4.3 Endometriosis and chronic pelvic pain

Endometriosis is characterized by the presence of endometrial-like tissue outside the uterus, affecting ~5–10% of reproductive-age women and is a leading cause of chronic pelvic pain and infertility (181). Its pathophysiology involves complex interactions between inflammatory processes, hormonal factors, and neural sensitization (181–183). Beyond local pelvic manifestations, endometriosis is increasingly recognized as having significant systemic dimensions, with affected women more susceptible to comorbid fibromyalgia, chronic fatigue syndrome, and mood disorders (183).

#### 4.3.1 Chinese herbal anti-inflammatory components' regulation of peripheral pain mechanisms

Chronic inflammation is a core feature of endometriosis pathophysiology, with endometriotic lesions producing various inflammatory mediators that promote pain, fibrosis, adhesion formation, and infertility (184). Local inflammation also sensitizes peripheral nociceptors, lowering pain thresholds and enhancing pain perception. Various Chinese herbal anti-inflammatory components may help address these peripheral aspects of endometriosis-associated pain (185, 186).

Resveratrol, a stilbenoid polyphenol found in Chinese herbs such as Polygonum cuspidatum, demonstrates significant anti-inflammatory and antioxidant properties relevant to endometriosis management (98). The anti-inflammatory actions of resveratrol in endometriosis involve multiple pathways, including inhibition of NF-κB signaling, reduction of pro-inflammatory cytokine production (particularly IL-1β, IL-6, and TNF-α), and inhibition of inflammatory enzyme activity (98, 187, 188). Additionally, resveratrol inhibits aromatase activity in endometriotic tissue, potentially reducing local estrogen production that drives lesion growth and inflammation (189, 190).

Beyond anti-inflammatory properties, resveratrol exhibits significant anti-angiogenic effects that may inhibit the development and progression of endometriotic lesions (98). Angiogenesis is a key process in endometriosis pathogenesis, as new blood vessel formation is necessary for lesion survival and growth. Functional imaging studies indicate that resveratrol treatment significantly reduces microvessel density and vascular endothelial growth factor (VEGF) expression in endometriotic lesions, resulting in reduced lesion size and pain improvement (189).

#### 4.3.2 Chinese herbal neuromodulation of central pain processing

Beyond peripheral inflammation, central sensitization of pain pathways is a key component of endometriosis-associated chronic pelvic pain (191). This process involves neuroplastic changes in the central nervous system, resulting in increased responsiveness of nociceptive neurons to normal and subthreshold stimuli (192). Various plant-derived compounds possess neuromodulatory properties that may help address these central aspects of endometriosis-associated pain, providing a more comprehensive approach to pain management (193–195). Beyond traditional anti-inflammatory and analgesic Chinese herbs, recent research has identified various Chinese herbal components that directly modulate central pain pathways. Tetramethylpyrazine, a key component in pain-relieving formulas, acts by activating descending inhibitory pain pathways, particularly through enhancing noradrenergic and serotonergic projections from the brainstem to the spinal cord (196, 197). Gastrodin from Gastrodia elata demonstrates significant central nervous system modulatory effects that may be particularly applicable to centrally sensitized pain in endometriosis (198–200). Gastrodin inhibits microglial activation, reduces catecholaminergic system hyperactivity in the spinal cord and brain, and normalizes NMDA receptor expression (198, 200).

## 5 Dietary supplements and functional foods: development and practical applications

### 5.1 Global development status of dietary supplements and functional foods

The market for dietary supplements and functional foods targeting women's reproductive health has experienced unprecedented growth over the past decade. In just 30 years, the U.S. dietary supplement market has evolved from several hundred products primarily consisting of vitamins, minerals, and select herbal extracts to >75,000 products today. The United States remains the world's largest dietary supplement market, having shown remarkable stability even during economic downturns. Historical data from the 2007–2010 National Health and Nutrition Examination Survey (NHANES) indicated that 49% of US adults reported using supplements (201, 202). The growth in this sector parallels rising healthcare costs and increasing prevalence of conditions affecting the female neuroendocrine-reproductive axis. Functional foods and supplements offer an accessible means for women to take proactive approaches to health maintenance, particularly in addressing symptoms associated with hormonal fluctuations throughout the reproductive lifespan. Importantly, these products often align with patient preferences for natural approaches and preventative strategies rather than solely relying on pharmaceutical interventions after symptoms develop (203, 204).

### 5.2 Current research and application of plant-based ingredients for women's reproductive health

The application of plant-derived compounds in women's health supplements has evolved considerably, with formulations becoming increasingly sophisticated and targeted. For menopausal health, products containing standardized extracts of soy isoflavones, red clover, and black cohosh dominate the market, with clinical evidence supporting their efficacy in managing vasomotor symptoms and potentially offering protective effects for cognitive health. These ingredients are commonly formulated as capsules, tablets, or incorporated into functional beverages and food products (205–208).

For premenstrual syndrome management, formulations containing chasteberry (Vitex agnus-castus), evening primrose oil, and St. John's wort have gained popularity, with varying levels of clinical evidence supporting their efficacy (209–213). Delivery systems for these ingredients range from traditional capsules to innovative formats such as functional teas, chocolates, and gummies, which may enhance compliance and address consumer preferences. Products targeting fertility and reproductive health often contain combinations of antioxidant-rich botanicals, including green tea extracts, resveratrol, and various adaptogens (66, 214, 215). These formulations aim to reduce oxidative stress, modulate inflammatory pathways, and optimize hormonal balance—all factors that contribute to reproductive health and fertility outcomes. The application of these ingredients spans multiple delivery formats, including specialized prenatal supplements, fertility-supporting beverage powders, and condition-specific formulations.

## 6 Future research directions and opportunities

### 6.1 Integrating advanced neuroimaging with Chinese herbal research

Functional magnetic resonance imaging (fMRI) and other advanced neuroimaging techniques provide unprecedented opportunities for studying the effects of Chinese herbal components on the neuroendocrine-reproductive axis (1, 216). These non-invasive methods track changes in brain activity and connectivity patterns, providing direct visualization of mechanisms underlying herbal effects (217, 218).

Each neuroimaging modality offers distinct insights into how plant compounds affect neural function: functional MRI reveals real-time brain activation and network connectivity changes (219, 220); diffusion tensor imaging (DTI) visualizes white matter structural integrity modifications that may reflect neuroplastic responses to herbal interventions (221, 222); and arterial spin labeling (ASL) measures cerebral blood flow changes that may underlie vascular mechanisms of herbal actions (223–225).

Multimodal imaging approaches are particularly valuable for herbal research because they can capture the multifaceted effects of plant compounds. For example, resveratrol supplementation not only enhances functional connectivity between the left posterior hippocampus and medial prefrontal cortex in older adults, but these connectivity improvements also correlate significantly with enhanced memory performance and decreased glycated hemoglobin (HbA1c) levels. These findings suggest a potential mechanism whereby resveratrol may support cognitive function in older adults by improving communication between key brain regions and optimizing metabolic function (226).

A promising application is tracking changes in prefrontal-hippocampal connectivity before and after phytoestrogen interventions in menopausal women, correlating these changes with cognitive improvements and hormone regulation (227–229). Advanced neuroimaging analytical methods could further elucidate how plant compounds affect broader neural networks, potentially revealing mechanisms beyond isolated regional effects and providing more comprehensive insights into their neuromodulatory actions (229, 230).

For endometriosis-related chronic pain, neuroimaging can identify predictive markers for herbal treatment response (231, 232). Neuroimaging could help visualize how traditional plant compounds affect central pain processing pathways in the brain (233, 234). Future research utilizing functional MRI could examine how herbal interventions modulate activity in key pain-related regions such as the anterior cingulate cortex and insula (235–238). Such studies may eventually reveal whether baseline functional connectivity patterns in pain processing networks could predict individual responses to herbal formulations, potentially enabling more personalized treatment approaches (239).

Future research should standardize neuroimaging protocols for herbal studies and incorporate advanced analytical approaches to capture the complex neural effects of multi-component herbal formulations (240, 241). The integration of neuroimaging with pharmacokinetic and genetic assessments could further elucidate individual variations in neural responses to plant-derived compounds, advancing personalized botanical interventions for women's neuroendocrine-reproductive health (242).

### 6.2 Clinical translation challenges and opportunities

Laboratory and preclinical studies on Chinese herbal active components in neuroendocrine-reproductive health require clinical translation. This process faces significant challenges, primarily the limited number of high-quality randomized controlled trials (RCTs) that incorporate objective biomarkers and neuroimaging endpoints (243, 244). Future clinical studies should adopt more rigorous methodologies with appropriate randomization, blinding, adequate sample sizes, and objective outcome assessments. Adaptive trial designs may be particularly suitable for herbal formulation research, allowing modifications based on preliminary results while maintaining scientific rigor. For example, studies evaluating menopausal cognitive interventions could adjust formulations based on participant responses, mirroring the personalized approach of traditional Chinese medicine pattern differentiation while maintaining methodological standards (245). Additionally, long-term studies examining the effects of Chinese herbs across different female life stages are needed. Longitudinal research tracking herbal interventions from puberty through post-menopause could assess preventive strategies for neuroendocrine-reproductive axis dysregulation, aligning with traditional Chinese medicine's preventive philosophy while generating valuable evidence for broader women's health strategies.

### 6.3 Innovative approaches in herbal formulation development

The application of Chinese herbal active components in functional foods represents a rapidly evolving field with significant market potential. Combining traditional wisdom with modern food technology can yield innovative products targeting neuroendocrine-reproductive health (114, 246). A key challenge is the limited bioavailability of many active components like curcumin and resveratrol, which often show promising results *in vitro* but fail to achieve therapeutic concentrations *in vivo* due to poor absorption, rapid metabolism, or limited solubility (247, 248).

#### 6.3.1 Advanced delivery systems for enhanced bioavailability

Nanotechnology offers promising solutions for overcoming bioavailability limitations of plant compounds. Nanoparticle-based delivery systems significantly enhance the solubility, stability, and cellular uptake of poorly bioavailable compounds (249). For instance, curcumin-loaded nanoparticles demonstrate substantially greater bioavailability compared to unformulated curcumin, with correspondingly enhanced therapeutic effects in both neurological and reproductive tissues (250–252). Polymeric nanoparticles, utilizing biodegradable materials like PLGA (poly lactic-co-glycolic acid), provide controlled release profiles particularly beneficial for compounds targeting the neuroendocrine-reproductive axis, where sustained therapeutic concentrations are often required for optimal efficacy (253, 254).

Liposomal encapsulation represents another effective strategy for enhancing bioavailability of plant compounds. These phospholipid vesicles can encapsulate both hydrophilic and hydrophobic compounds, protecting them from degradation while facilitating cellular uptake through membrane fusion or endocytosis (255). Liposomal formulations of resveratrol have demonstrated markedly increased bioavailability and significantly enhanced neuroprotective effects in cognitive studies (256). For women's reproductive health applications, liposomal delivery systems can be designed with specific surface modifications to target reproductive tissues, enhancing local concentration of therapeutic compounds while minimizing systemic exposure.

Micelle-based delivery systems utilize amphiphilic molecules that spontaneously form nano-sized core-shell structures in aqueous environments, encapsulating hydrophobic compounds within their cores (257). These systems dramatically increase the apparent water solubility of compounds like EGCG and curcumin (258–260). Mixed polymeric micelles combining different polymers can be particularly effective for delivering multiple plant compounds simultaneously, supporting the synergistic effects characteristic of traditional herbal formulations while improving their bioavailability profiles.

#### 6.3.2 Bioenhancement strategies for plant compounds

Beyond delivery systems, specific bioenhancement strategies can significantly improve the absorption and effectiveness of plant compounds. Co-administration with natural absorption enhancers represents a traditional approach now validated by modern science. Piperine from black pepper inhibits UDP-glucuronosyltransferase and hepatic aryl-hydrocarbon hydroxylase, significantly reducing the metabolism of compounds like curcumin and resveratrol (261–264). Studies demonstrate that co-administration of piperine substantially increases curcumin bioavailability, potentially enhancing its therapeutic effects across various applications (262, 263).

Phytosomal technology, combining plant compounds with phospholipids to form molecular complexes with improved lipophilicity, offers another effective enhancement strategy. Phytosomes demonstrate superior bioavailability compared to simple herbal extracts while maintaining the natural origin preferred by many consumers (265–267).

Self-emulsifying drug delivery systems (SEDDS) spontaneously form fine oil-in-water emulsions upon mild agitation in the gastrointestinal tract, significantly enhancing the solubility and absorption of lipophilic compounds (268–270). These systems are particularly valuable for enhancing the bioavailability of fat-soluble plant compounds like phytoestrogens and certain polyphenols, which otherwise show limited absorption. SEDDS formulations can be easily incorporated into soft gel capsules or functional food formats, supporting patient compliance while significantly enhancing therapeutic efficacy (269, 271).

Future research should optimize these technologies for women's health applications, such as developing phytoestrogen delivery systems capable of crossing the blood-brain barrier to enhance cognitive protection. Functional food formulations should also consider life-cycle approaches, targeting specific stages and transitions in female physiology. For example, formulations based on classical Chinese herbal prescriptions could be adapted for different age groups—adding menstruation-regulating herbs for young women, antioxidant components for middle-aged women, and cognitive support components for older women, each with optimized bioavailability enhancement strategies appropriate for their target compounds and tissues.

### 6.4 Interdisciplinary integration for future research

The fusion of Chinese herbal research with neuroendocrinology, gynecology, and nutritional science represents a particularly promising direction. This interdisciplinary integration can generate new therapeutic paradigms combining traditional wisdom with cutting-edge science (272, 273). To advance research in this field, international collaborative platforms integrating diverse expertise should be established. These platforms should bring together traditional Chinese medicine practitioners, neuroscientists, endocrinologists, gynecologists, and nutritionists to design and execute comprehensive research projects. Standardized methods are essential for generating comparable and reproducible results, including standardized analytical protocols for herbal extracts and active components, comprehensive assessment frameworks for neuroendocrine-reproductive axis function, approaches that integrate traditional diagnostics with modern biomarkers, and quality control standards for herbal formulations (274). Natural language processing and machine learning technologies offer new opportunities to analyze ancient Chinese medical texts, potentially identifying herbal patterns for specific gynecological conditions that may have been overlooked by modern research. These data-driven discoveries can guide the development of novel therapeutic strategies, bridging traditional knowledge with contemporary science (272).

### 6.5 Regulatory considerations and global application

The global application of Chinese herbal products faces regulatory challenges, with different regions having varying classifications and requirements. Developing harmonized regulatory frameworks that recognize traditional evidence while ensuring safety and efficacy is essential for promoting the legitimate use of Chinese herbal products internationally (275). Future efforts should include developing international quality standards for Chinese herbal products, establishing innovative clinical trial designs for evaluating herbal formulations, creating methodological frameworks that integrate traditional evidence with modern clinical data, and training healthcare professionals to incorporate Chinese herbs into women's healthcare (272, 276, 277). These regulatory and educational advances will accelerate the translation of Chinese herbal research into clinical practice and consumer products, expanding access to these potential therapeutic approaches.

## 7 Conclusion

Plant-derived bioactive compounds from Chinese medicinal plants demonstrate significant advantages in anti-inflammatory, antioxidant, neuroregenerative, and mitochondrial protective functions relevant to neuroendocrine-reproductive health. This review systematically examined how these compounds simultaneously modulate neurological and reproductive systems through multiple pathways, providing evidence for their applications in menopausal cognitive function, premenstrual syndrome, polycystic ovary syndrome, and endometriosis-related pain management. Future research should focus on developing optimized clinical trial designs specifically adapted for complex herbal formulations, such as adaptive designs that allow for personalization while maintaining scientific rigor. Establishing multi-center collaborative studies with standardized protocols will generate more robust evidence across diverse populations, while investigating integrative therapeutic strategies that combine plant-based compounds with conventional pharmaceuticals could enhance efficacy and reduce side effects in conditions resistant to single-modality treatments. Additionally, applying personalized medicine approaches using advanced -omics technologies to identify biomarkers that predict individual responses would allow for more precise herbal interventions. Advanced functional neuroimaging and molecular research increasingly validate the regulatory mechanisms of these compounds on the female neuroendocrine-reproductive axis. As core components of dietary supplements and functional foods, Chinese herbal ingredients offer substantial market potential while addressing contemporary neurological health concerns, providing evidence-based options for improving women's neuroendocrine-reproductive health globally.
